# Attentional Engagement for Pain-Related Information among Individuals with Chronic Pain: The Role of Pain Catastrophizing

**DOI:** 10.1155/2018/6038406

**Published:** 2018-12-02

**Authors:** J. E. Lee, S. H. Kim, S. K. Shin, A. Wachholtz, J. H. Lee

**Affiliations:** ^1^Department of Psychology, Chung-Ang University, Seoul 06974, Republic of Korea; ^2^Department of Psychology, University of Colorado-Denver, Denver, CO 80204, USA

## Abstract

Although the evidence of the attentional bias of chronic pain individuals toward pain-related information is established in the literature, few studies examined the time course of attention toward pain stimuli and the role of pain catastrophizing on attentional engagement toward pain-related information. This study examined the time course of attention to pain-related information and the role of pain catastrophizing on attentional engagement for pain-related information. Participants were fifty young adult participants with chronic pain (35% male, 65% female; M = 21.8 years) who completed self-report questionnaires assessing pain catastrophizing levels (Pain Catastrophizing Scale (PCS)), depression (the Center for Epidemiologic Studies Depression Scale (CES-D)), anxiety (State-Trait Anxiety Inventory (STAI)), and pain disability (the Pain Disability Index: (PDI)). Attentional engagements to pain- and anger-related information were measured by the eye tracker. Significant interaction effects were found between (1) time and stimulus type for pain-related information (*F* (5, 245) = 11.55, *p* < 0.001) and (2) bias scores and pain catastrophizing (*F* (1, 48) = 6.736, *p* < 0.05). These results indicated that the degree of increase for pain bias scores were significantly greater than anger bias scores as levels of pain catastrophizing increased. Results of the present study provided the evidence for the attentional bias and information processing model which has clinical implications; high levels of pain catastrophizing may impair individuals' ability to cope with chronic pain by increasing attentional engagement toward pain-related information. The present study can add knowledge to attentional bias and pain research as this study investigated the time course of attention and the role of pain catastrophizing on attentional engagement toward pain-related information for adults with chronic pain conditions.

## 1. Introduction

Meta-analytic studies show the evidence for pain attentional bias among chronic pain individuals [1, 2]; however, this evidence is based on studies that measure indirect and covert attention such as the dot-probe task or cueing task [3]. Most recently, eye tracking methods have been introduced to measure attentional process more directly. Eye tracking methodology is a continuous measure that allows researchers to observe attentional patterns in different phases of attention [4]. In spite of this improvement in eye tracking, only a few studies have examined the time course of the attentional process to pain stimuli. For instance, Priebe et al. [5] examined the time course of gaze behaviours toward pain faces using the eye tracker. They found healthy adults initially preferred pain faces but this attentional preference declined over the course of time. The authors argued that this decline in attentional preference could be interpreted as either a shift to attentional avoidance from pain faces or the loss of relevance for pain faces over time. They speculated that an attentional pattern to pain stimuli might have been shown more clearly if stimuli were presented longer than 2,000 ms. Despite this research in healthy adults, little is known about the time course of the attentional process toward pain stimuli among individuals with chronic pain.

Although a meta-analysis [2] reported nonsignificant effects of psychological factors on attentional bias toward pain-related information, fMRI research investigating the mechanism of pain experience revealed that pain catastrophizing influences pain perceptions by increasing an individual's attention toward pain-related stimuli [6]. According to the attentional bias and information processing model [7, 8], individuals who exaggerate the threatening values of pain stimuli may increase their attention toward pain-related information. However, only a few studies (e.g., [9]) investigated the role of pain catastrophizing on the attentional engagement of adults with chronic pain toward pain-related information by using the eye tracker. Vervoort et al. [9] found that high catastrophizing, chronic pain individuals showed a preference toward both pain and neutral facial expressions.

The current study expanded on previous studies by investigating the time course of attentional engagement toward pain facial expressions among young adults with chronic pain over the course of 3,000 ms. Further, the present study compared attentional engagement toward pain facial expressions with that of angry facial expressions when examining the time course of attentional engagement as well as the total attentional process. This method was used to determine whether attentional engagement to pain-related information is a natural attentional response to threat-related information (i.e., angry facial expressions) or a specific response to pain-related information. A previous study [5] measured attentional engagement to pain as well as other threat-related information (such as angry faces); however, they did not directly compare pain bias scores with anger bias scores.

Lastly, the present study examined how the attentional engagement toward pain-related information differs depending on psychological factors, particularly pain catastrophizing levels. The present study proposed that (1) attentional engagement toward pain-related information would initially increase and be maintained throughout the course of attention and (2) attentional engagement toward pain-related information would be influenced by pain catastrophizing levels but the same pattern would not occur for anger-related information.

## 2. Materials and Methods

### 2.1. Participants

Participants were adults who were diagnosed with a chronic pain condition. They were recruited from university psychology courses as well as online advertisements from universities located in Seoul, Korea. All participants were initially screened using a questionnaire to assess the presence of chronic pain, the diagnosis, location(s) of most significant pain, pain intensity, pain duration, and pain frequency (per month). The present study utilized ICD definition of chronic pain (pain that lasts more than 3 months) [10]. Inclusion criteria for the present study were (1) the presence of chronic pain diagnosis, (2) pain duration more than 3 months, and (3) self-rating average pain above 5 on a 10-point scale. Exclusion criteria were (1) inability to read Korean, (2) inability to clearly see pictures presented on the screen 60 cm away without corrective lenses, (3) having had LASIK or LASEK surgery, and (4) having cataracts or glaucoma.

As the power analysis of repeated measure ANOVA with within-subjects indicated that the minimum sample size should be 28 (effect size = 0.25, alpha error probability = 0.05, power = 0.80), we increased the group to 50 to account for a possibility of a maximum 10% dropout rate. A total of 50 young adults (male = 17, female = 33) with a mean age of 21.80 years (SD = 2.06), currently experiencing chronic pain, were recruited for the present study. All participants completed informed consent procedures and were informed that they can terminate the experiment at any time. The study was approved by the ethics committee of Chung-Ang University (IRB # 1041078-201609-HRSB-167-01).

### 2.2. Measures

Demographic information and pain-related questions were included in the survey. Participants were asked to answer the questions related to their age, gender, smoking status and an average number of cigarettes per week, and drinking status and an average number of drinks per week. Pain-related questions included the following items: (1) pain duration, (2) pain level during the past three months, and (3) days experiencing pain per month. Regarding pain intensity, participants rated on an 11-point numeric rating scales (NRS), ranging from 0 (no pain) to 10 (most extreme pain).

Pain Catastrophizing Scale [11] includes 13 items measuring the three components of pain catastrophizing: rumination (e.g., “I can't seem to keep it out of my mind”); magnification (e.g., “I wonder whether something serious may happen”); and helplessness (e.g., “There is nothing I can do to reduce the intensity of pain”). This scale has been translated and standardized in Korean population [12]. Participants rated in reference to a previous pain event on a 5-point Likert scale ranging from 0 (not at all) to 4 (always). Cronbach's alpha of K-PCS was 0.93 [12]. In the present study, Cronbach's alpha of K-PCS was 0.91.

The Pain Disability Index [13] that was translated into Korean [14] was utilized for the present study. PDI measures the degree of disturbance caused by pain in daily life. PDI consists of 7 items on 7 categories of life domains (i.e., home, entertainment, social, occupation, sex life, self-management, and life maintenance). Each item is rated on an 11-point Likert scale ranging from 0 (no disability) to 10 (worst disability). Higher score indicates more pain interference with life. In the present study, Cronbach's alpha of PDI was 0.89.

The Center for Epidemiologic Studies Depression Scale (CES-D [15]) was translated and standardized by Chon, Choi, and Yang [16]. CES-D is a self-report 20-item questionnaire assessing depressive symptoms. Participants were asked how often they felt a certain way over the past week, with four response options ranging from 0 (rarely) to 3 (all the time). Cronbach's alpha of KCES-D was 0.91 [16], and Cronbach's alpha of KCES-D in the present study was 0.92.

State-Trait Anxiety Inventory (STAI) [17] was administered to measure state and trait anxiety. STAI is a 40-item self-report questionnaire rated on a four-point Likert scale ranging from 1 (not at all) to 4 (very much so). This scale is composed of two subscales: state anxiety (20 items) and trait anxiety (20 items). The present study used trait anxiety subscle of the STAI Korea which was translated and standardized by Kim and Shin [18], and they reported internal consistency of 0.87 with Korean college student population. The current study obtained internal consistency of 0.87 for trait anxiety.

### 2.3. Stimulus Materials

Visual stimuli used in this study included pictures of 8 adult faces (4 males and 4 females). Visual stimuli displaying anger and neutral expressions were obtained from the Korea University Collection (KUFEC), which were validated in the previous study [19]. Pictures displaying pain expressions were created by inserting prototypical action units associated with pain (e.g., narrowed eyes, upper lip raiser, lowering eye brows, and cheek raiser; [20–22]) on pictures extracted from the Korea University Collection.

All colored images extracted were converted into monochrome images to decrease the effects of emotions associated with color [5]. Furthermore, as a way of diminishing distractions associated with hairstyles, all photos were resized so that only the faces were shown in all photos [9]. Because pain expression photos have not been validated in previous studies, 15 psychology graduate student judges (5 males, 10 females) and 45 undergraduate student judges (26 males, 19 females) determined how reliable pain photos were using an 8-point scale (0: not at all relating to pain to 7: very much relating to pain) in the present study. Pain expression photos were converted into monochrome images and resized prior to conducting the validity check. The mean rating of pain facial expressions (M = 5.67, SD = 0.51) was significantly different from that of neutral facial expressions (M = 2.11, SD = 0.41; *t* (22) = −20.346, *p* < 0.001). The angry facial expressions were also rated using an 8-point scale (0: not at all relating to anger to 7: very much relating to anger). The mean rating of angry facial expressions (M = 7.65, SD = 1.13) was significantly different from that of neutral facial expressions (M = 1.94, SD = 0.54; *t* (14) = 12.873, *p* < 0.001). Intensity of pain facial expressions (M = 7.029, SD = 0.641) was slightly lower than that of angry facial expressions (M = 7.428, SD = 0.853). However, there was no significant difference between the intensity of pain facial expressions and that of anger facial expressions (*t* (22) = −1.287, *p*=0.212).

To distinguish whether a participant was biased toward a high-arousal stimulus by threat-related stimuli rather than a degree of pain, photos depicting angry expressions that corresponded to the photos of faces displaying pain expressions were obtained from KUFEC. In the case of photos displaying neutral expression, KUFEC's photographs corresponding to the selected photos of pain facial expression were extracted. Using these pictures, we created a total of 64 pairs of pictures that were horizontally aligned. Among 56 slides, a series of three different pairs were generated. These pairs consisted of a neutral face combined with a neutral face (filler slides), a pain face, or an angry face. Pairs were compiled twice such that the neutral expression appeared on the left side as often as it did on the right.

Pairs of photos were presented side by side on the gray background [23]. The eye movements during the gazing at the stimuli were measured by the Tobii TX300 eye tracker (Tobii Technology, Stockholm, Sweden). This system consists of a 22-inch LCD monitor with a camera that is attached to the bottom of the screen. The stimuli were presented in a strict order: a central fixation point (1,000 ms), facial expression stimulus (3,000 ms), and the blank screen (1,000 ms). All experiments were conducted at the default sampling rate of the eye tracker and at the optimum distance between the eye tracker and the subject. For TX300, the sampling rate was 300 Hz and the distance 65 cm (27″). Precision is calculated as root-mean-square (RMS) of successive samples. The stimulus paradigm for the measurement of attentional bias was based on previous studies using eye movement tracking equipment [24, 25]. After sitting on a chair; the participants were asked to hold their postures to minimize head movement and stared at the photo stimuli through a monitor, which was 60 cm away. The size of the individual photographic stimuli presented on the monitor screen was 55 cm (width) × 24 cm (length) with a resolution of 1920 × 1080 pixels, and the viewing angle was 38°. Gaze accuracy and precision are typically measured in degrees of visual angle. One-degree accuracy corresponds to an average error of 12 mm (0.47″) on a screen at a distance of 65 cm (27″). All measurements are done at the default sampling rate of the eye tracker and at the optimum distance between the eye tracker and the subject. For TX300, the sampling rate was 300 Hz and the distance 65 cm (27″). This system defined fixation if participants' gaze did not deviate from a defined area of interest (AOI), which was the entire photo, for at least 100 ms. In order to measure attentional engagement to pain- and anger-related information, total gaze duration was measured [26]. Total gaze duration was defined as the averages of total fixation durations within an AOI either during the entire time (3000 ms) or during the specified time (e.g., 0–500 ms, 1000 ms–1500 ms, 2500 ms–3000 ms, etc.) [5, 27]. We selected the 3,000 ms presentation time in order to observe maintained attention [9].

### 2.4. Procedure

After their arrival, participants completed the consent form. Prior to the computer task, participants were told that they would watch various pictures as if they were watching TV or looking at a picture album. Participants were instructed to sit comfortably and minimize their movements while they followed a red dot on the screen during the calibration process to measure eye movements precisely. When the calibration process was complete, participants were asked to freely view the pictures presented on the screen during the free-viewing task for 10 minutes. During the trial, participants were initially instructed to stare at the blank black screen and then stare at the white cross before the pictures appeared on the screen. Total 64 stimulus pairs were presented. To eliminate position effects, the positions of neutral pictures were randomized during trials. After the completion of the free-viewing task, participants were asked to fill out questionnaires.

### 2.5. Statistical Analysis

In order to investigate the time course of attentional engagement toward threat-related information (pain and anger), repeated measurement analyses of variance (ANOVAs) with the within‐factors “time” (0–500 ms vs. 500–1000 ms vs 1000–1500 ms vs. 1500–2000 ms vs. 2000–2500 ms vs. 2500–3000 ms), “stimulus type” (neutral vs. pain or anger expressions) were performed using SPSS. Repeated measurement analyses of covariance (ANCOVAs) was also conducted with the within-factor “bias scores” (pain bias score vs. anger bias score) and “covariate” (pain catastrophizing) as a direct comparison between the attentional bias of pain and that of anger-related information and as a way to explore how pain catastrophizing levels influence on the attentional bias to pain and anger expressions. Bias scores were obtained by subtracting the total gaze durations of neutral faces from the total gaze durations of either pain- or anger-related information. Paired *t*-tests were conducted as post hoc tests to clarify the interaction effects of ANOVAs.

## 3. Results and Discussion

### 3.1. Participant Characteristics

Table 1 displays descriptive statistics of demographic variables (i.e., age, gender, drinking habits, and tobacco use) as well as pain-related and psychological variables (i.e., pain duration, pain days per month, average pain intensity in past 3 months, pain diagnosis, pain catastrophizing level, pain disability, depression, and trait anxiety) for the sample.

Intercorrelation analyses were conducted between psychological variables (i.e., pain catastrophizing, depression, and trait anxiety), pain-related variables (i.e., frequencies of chronic pain per month, average pain intensity, pain intensity during past 3 months, and total months for participants experienced chronic pain), and dependent variables (i.e., total gaze duration to pain and angry faces). In summary results revealed that pain catastrophizing was not also significantly associated with pain frequency (*r* = 0.143, *p*=0.323) and total months experiencing chronic pain (*r* = −0.051, *p*=0.723). However, pain catastrophizing was significantly associated with average pain intensity (*r* = 0.288, *p* < 0.05), 3-month pain intensity (*r* = 0.358, *p* < 0.05), pain disability (*r* = 0.516, *p* < 0.001), depression (*r* = 0.345, *p* < 0.05), and trait anxiety (*r* = 0.483, *p* < 0.001). Pain catastrophizing was also significantly correlated with total gaze durations toward pain-related information (*r* = 0.355, *p* < 0.05) but not with total gaze duration toward anger-related information (*r* = 0.232, *p*=0.105). In summary, results indicated that high levels of pain catastrophizing were associated with higher levels of total gaze durations toward pain-related information, higher levels of pain intensity, and the higher level of distress and pain‐related adjustment difficulties (e.g., depression, trait anxiety, and pain disability).

Table 2 shows ANOVA and chi-squared test results for the three types of pain diagnosis group (e.g., head pain, back pain, and other types of chronic pain). Results indicated that there were no significant differences among the three groups on demographic and pain-related variables (i.e., age, pain intensity, and pain disability), psychological variables (i.e., pain catastrophizing, anxiety, and depression), and dependent variables (i.e., total gaze duration toward pain- and anger-related information). In order to explore the effect of gender on pain catastrophizing, total fixation toward pain-related information, and total fixation bias score, the correlation analyses and *t*-test were performed. Results indicated that gender was not significantly correlated with any of the variables mentioned above. *T*-test result results also revealed that no significant gender differences on pain catastrophizing scores, total gaze duration to pain-related information, and bias score for total fixation. Based on these results, no additional analyses were conducted.

### 3.2. Time Course of Gaze Durations

Repeated measure ANOVAs with within factors “time” (0–500 ms vs. 500–1000 ms vs. 1000–1500 ms vs. 1500–2000 ms vs. 2000–2500 ms vs. 2500–3000 ms) and “stimulus type” (neutral vs pain or anger expressions) were performed separately for the pain-neutral pairs and the anger-neutral pairs. Significant effects were also found on time (*F* (5, 245) = 66.38, *p* < 0.001, *η*^*2*^ = 0.575), stimulus type (*F* (1, 49) = 56.04, *p* < 0.001, *η*^*2*^ = 0.534), and the interaction between time and stimulus type (*F* (5, 245) = 11.55, *p* < 0.001, *η*^*2*^ = 0.191) for pain and neutral pairs (Table 3). For anger and neutral pairs, significant effects were also found on time (*F* (5, 245) = 49.90, *p* < 0.001, *η*^*2*^ = 0.505), stimulus type (*F* (1, 49) = 30.19, *p* < 0.001, *η*^*2*^ = 0.381), and the interaction between time and stimulus type (*F* (5, 245) = 5.65, *p* < 0.001, *η*^*2*^ = 0.103; Table 4).

**Figure 1:**
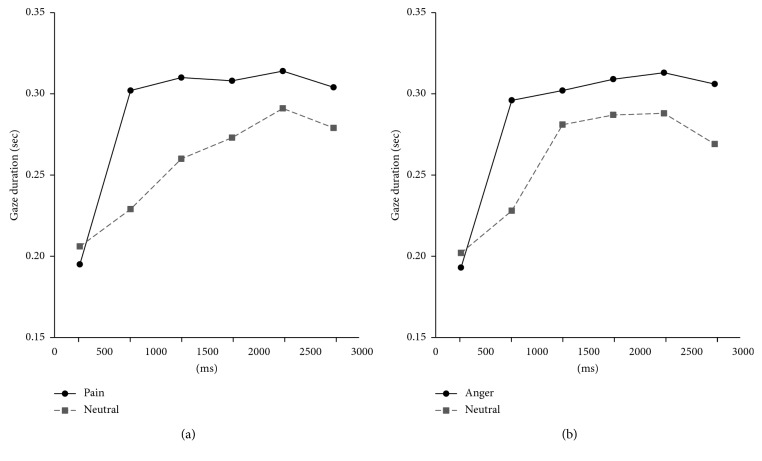
Means of gaze durations for pain and anger faces compared with neutral faces over six times during stimulus presentation.

Figure 1 also shows that the discrepancies between gaze durations of pain- and anger-related information and those of neutral expressions increased during the middle phase of attention (i.e., 500 ms–1,000 ms [4]) and maintained to the later stage of attention (i.e., 1000 ms–3000 ms). In order to further examine the significant interaction effects between time and stimulus type, paired *t*-tests were performed as a post hoc tests (Bonferroni-corrected *α'* = 0.008) to compare the gaze durations of pain- and anger-related stimuli with those of neutral stimuli on six different time points (i.e., 0–500 ms, 500 ms–1,000 ms etc., Table 5). For pain-related information, results revealed that participants gazed at photos displaying pain expressions significantly longer than neutral expressions at time 2 (*t* (49) = 5.491, *p*=0.000, 95% CI of *t* (.046, 0.099)), time 3 (*t* (49) = 5.627, *p*=0.000, 95% CI of *t* (0.032, 0.067)), time 4 (*t* (49) = 3.891, *p*=0.000, 95% CI of *t* (0.017, 0.053)), time 5 (*t* (49) = 3.365, *p*=0.001, 95% CI of *t* (0.009, 0.037)), and time 6 (t (49) = 3.322, *p*=0.002, 95% CI of *t* (0.010, 0.040)). For anger-related information, participants gazed at photos displaying anger expressions significantly longer than neutral expressions at time 2 (*t* (49) = 5.054, *p*=0.000, 95% CI of *t* (0.041, 0.095)) and time 6 (*t* (49) = 3.455, *p*=0.001, 95% CI of *t* (0.015, 0.058)).

In summary, results showed a different time course in gaze durations for pain-related information compared to anger-related information. It appeared that discrepancies between emotion and neutral expressions were larger throughout the course of time for pain facial expressions compared to anger facial expressions.

### 3.3. Bias Scores and Psychological Variables including Pain Catastrophizing as Covariates

A repeated-measure ANCOVA was performed on bias scores (pain bias score vs. anger bias score) and covariates (pain catastrophizing, depression, trait anxiety, and state anxiety). As shown in Table 6, there was no significant main effect but a significant interaction effect between bias scores and pain catastrophizing was observed (*F* (1, 48) = 6.736, *p* < 0.05, *η*^*2*^ = 0.123). Interactions between bias scores and other psychological variables were not found to be significant.

**Figure 2:**
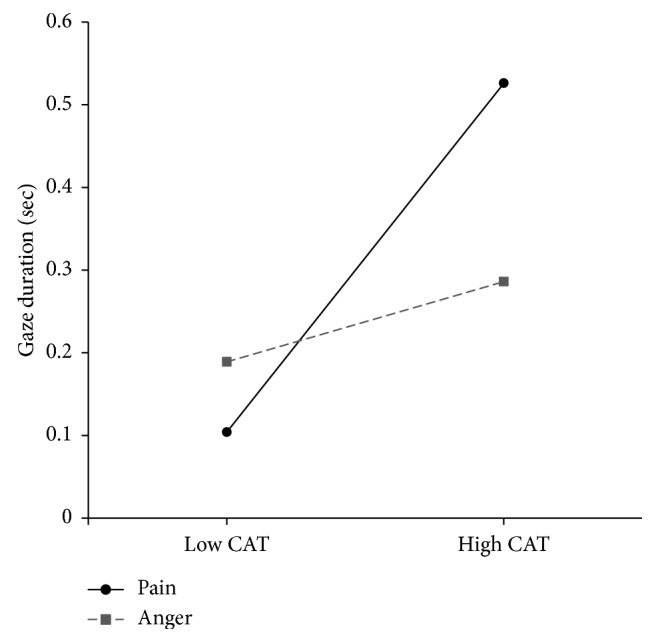
Plotting total gaze durations for pain- and anger-related information depending on the degrees of pain catastrophizing. *Note.* High CAT = high catastrophizing group which contains participants whose pain catastrophizing scores are 1 SD above the mean; low CAT = low catastrophizing group which contains participants whose pain catastrophizing scores are 1 SD below the mean.

As shown in Figure 2, the interaction effect between pain catastrophizing and bias score was plotted at two levels of pain catastrophizing (1 SD below the mean and 1 SD above the mean of pain catastrophizing). This plot showed that the degree of increase for the pain bias score was significantly greater compared to the increase rate for the anger bias score as levels of pain catastrophizing increased.

## 4. Discussion

The present study investigated the time course of attentional engagement toward pain-related information among young adults diagnosed with chronic pain conditions. We hypothesized that attentional engagement would initially increase and be maintained toward pain-related information over the course of time, and the present study supported this hypothesis. Consistent with the previous study [5], attentional engagement toward pain- and anger-related information radically increased during the middle stage of attention (i.e., time 2: 500 ms–1,000 ms). These results may due to human's natural tendencies to rapidly increase their attention toward emotional responses when participants become consciously aware of stimuli [5]. Schoth et al. [1] explained that emotional expressions usually capture attention as those emotional expressions are socially and biologically important. Particularly, facial expressions that are potentially interpreted as threatening will be more likely to receive attention until individuals evaluate those stimuli as nonthreatening. This may be the reason our results differ from results of Pribe's study [5]. Healthy individuals with no pain in Pribe's study [5] might decrease their attention to pain-related information during the later stage of their attention because pain facial expressions were not relevant to their personal experiences. In Pribe's study [5], the reduction of attentional engagement only occurred for pain facial expressions but not for other emotional expressions such as anger and happy facial expressions. In the present study, attention engagement toward pain-related information did not decline throughout the remaining course of attention because participants with chronic pain might have difficulty in disengaging from pain-related information due to relevancy or threatening values of pain facial expressions.

Examining the time course of attentional engagement more closely demonstrated distinct attentional preference patterns toward pain-related information compared to neutral expressions. Except for the early stage of attention (i.e., 0–500 ms), participants in the present study gazed significantly more at pain facial expressions than neutral facial expressions throughout the entire course of attention. This pattern did not occur for anger-related information. These results are consistent with the concept that attentional bias in chronic pain is a conscious process rather than a preattentive process similar to attentional bias in depression [2].

Supporting our second hypothesis, we found that participants with higher levels of pain catastrophizing engaged their attention significantly more toward pain-related information compared to those with lower levels of pain catastrophizing. This result may suggest that chronic pain sufferers who endorse high levels of pain catastrophizing increase their attention and maintain their attention to pain-related information because they either exaggerate threatening values of pain stimuli or engage in repetitive negative thoughts related to pain. Difficulty in disengaging from pain-related information may impair individuals' ability to cope with chronic pain more severely when individuals endorse high levels of pain catastrophizing. In other words, dwelling on pain-related information may lead them to repetitive thinking about pain and maladaptive appraisals of their situations, and therefore, it can reduce flexibility in coping [28]. Furthermore, their negative expectations about their situations may result in an underestimation of their personal efficacy. This may reduce their effort of coping with chronic pain and activity and, as a result, can increase psychological distress among individuals with chronic pain who report high levels of pain catastrophizing. For the present study, results revealed that high levels of pain catastrophizing were associated with more disability in participants' daily lives (e.g., social functioning, family functioning, job ability, and sexual functioning) and higher levels of pain and psychological distress (e.g., depression and trait anxiety).

Second hypothesis was not supported for anger-related information. Additionally, other psychological variables such as depression and anxiety did not interact with attentional engagement toward pain-related information as pain catastrophizing did. These results underscore the importance of pain catastrophizing in understanding attentional bias to pain-related information for individuals with chronic pain.

The results of the present study have clinical implications; current findings are particularly pertinent to developing effective attentional bias modification program (ABM) for chronic pain individuals. Utilizing the most relevant stimuli is crucial for ABM training, and the result of the present study showed the relevancy and importance of pain facial expressions compared to anger expressions. The present study could add more knowledge to ABM training literature as the results suggested that ABM training that utilizes pain facial expressions can be particularly useful for individuals with high level of pain catastrophizing. Mental health clinicians and health care professionals who treat chronic pain patients should be mindful that pain catastrophizing plays an important role by facilitating maintenance of attention to pain-related stimulus; these conscious attentional processes influence individuals' coping processes negatively. Therefore, the interventions should target conscious and control levels of the attentional process for chronic pain sufferers with high levels of pain catastrophizing. For instance, the cognitive modification of an attentional bias program can assist high-catastrophizing individuals to shift their attention to information that is not related to pain by modifying their attentional processes through computer-based cognitive training. Psychotherapies such as cognitive behavior therapy (CBT) and acceptance commitment therapy (ACT) can also help high-catastrophizing chronic pain sufferers reinterpret their situations and develop coping skills to reduce rumination and catastrophizing thinking related to chronic pain.

Despite the contributions of the present study, there are limitations that need to be addressed. First, participants for the present study only included a nonclinical population with a variety of chronic pain diagnoses. The present study found no significant differences on dependent variables, demographic variables, and psychological variables among different types of pain diagnosis groups (e.g., chronic head pain, chronic back pain, and other types of chronic pain); however, these results should be interpreted with caution due to small sample sizes for each group. Future studies should benefit from including clinical sample with a specific chronic pain diagnosis such as chronic back pain diagnoses or chronic headache diagnoses and also investigating how different types of chronic pain diagnoses (e.g., chronic head pain vs. chronic back pain) responded to different types of visual pain-related stimuli (e.g., pain faces vs. a photo of person who is displaying pain behaviours). Second, as the present study was conducted at a university located in Seoul, Korea, the interpretation of results in the present study should be generalized with caution to other geographic locations and cultures. Third, a previous study [29] found that participants with high levels of fear of pain were hypervigilant toward all stimuli including emotional and neutral stimuli. Additional research that examines how fear of pain interacts with pain catastrophizing when investigating attentional bias toward pain-related information is warranted. Lastly, although the present study calculated pain and anger bias scores and used those two scores as a way to compare attentional patterns of pain-related information with those of anger-related information, the direct comparisons between pain-related information and anger-related information may provide valuable information for the specificity of pain-related information regarding attentional engagement. This could be done in the future study by including pain and anger face pairs for the trials.

## 5. Conclusion

The present study investigated the time course of attentional bias to pain-related information as well as the role of pain catastrophizing on attentional engagement toward pain-related information. The results of the present study underscored the important role of pain catastrophizing in the attentional engagement biases for pain-related information. Pain expressions tend to be more ambiguous compared to anger expressions [1] so psychological factors such as pain catastrophizing may have a more significant impact on attentional engagement toward pain expressions compared to anger expressions. Given that relatively few pain and attentional bias studies have focused on the role of pain catastrophizing with individuals with chronic pain, the results of the present study can add valuable information as for how psychological factors particularly pain catastrophizing can influence on attentional patterns to pain-related information. This information can be used when clinicians and educators develop interventions and/or education programs for adults with chronic pain in community and college settings.

## Figures and Tables

**Table 1:** Descriptive statistics of demographic information and pain-related and psychological variables.

Variables	Mean (SD)
Age	21.80 (2.06)
Gender	
** **Male	34%
** **Female	66%
Smoking	
** **Yes	12%
** **No	88%
Drinking	
** **Yes	90%
** **No	10%
Chronic pain durations (months)	46.62 (29.04)
Average pain intensity in past 3 months (1–10)	6.40 (1.23)
Pain days per month	16.42 (7.40)
Pain disability index	30.72 (13.38)
Pain catastrophizing	19.98 (8.83)
Depression	21.74 (10.98)
Trait anxiety	47.60 (9.56)
Chronic pain diagnosis	*N* = 40
** **Cervical disc	4
** **Scoliosis	3
** **Spinal disc herniation	5
** **Spine compression fracture	1
** **Back pain	3
** **Migraine	10
** **Myofascial pain syndrome	1
** **Temporomandibular joint disease	4
** **Lumbar disc	1
** **Tension headache	6
** **Osteoarthritis	2

**Table 2:** Descriptive statistics of demographic information and pain-related and psychological variables by three types of pain diagnosis groups.

Variables	Mean (SD)			*F*/χ^2^
	Back pain (*N* = 17)	Head pain (*N* = 17)	Other pain (*N* = 16)	
Age	21.64 (1.65)	21.64 (2.24)	22.00 (2.25)	0.179
Gender				1.705
** **Male	42.90%	14.30%	40.90%	
** **Female	57.10%	85.70%	59.10%	
Chronic pain durations (months)	39.14 (31.06)	46.79 (33.41)	51.27 (24.88)	0.739
Average pain intensity in past 3 months (1–10)	6.50 (1.23)	6.71 (1.14)	6.14 (1.28)	1.011
Pain days per month	14.29 (6.11)	15.57 (8.58)	18.32 (7.1)	1.424
Pain disability index	31.86 (11.99)	33.50 (13.95)	28.23 (13.98)	0.727
Pain catastrophizing	19.50 (9.92)	20.00 (8.92)	20.27 (8.46)	0.032
Depression	22.07 (8.67)	23.43 (12.35)	20.45 (11.70)	0.313
Trait anxiety	47.64 (8.58)	48.43 (8.59)	47.05 (11.02)	0.086
Total gaze duration to pain	1.180 (0.304)	1.269 (0.293)	1.281 (0.266)	0.584
Total gaze duration to anger	1.148 (0.285)	1.222 (0.285)	1.280 (0.242)	1.044

**Table 3:** Summary of repeated measure ANOVA for pain-neutral pairs—time and stimulus type as within factors on gaze duration to pain-related information.

	*F*	*p*	*η* ^2^
Time	66.382	0.000^∗∗∗^	0.575
Stimulus type	56.041	0.000^∗∗∗^	0.534
Time ∗ stimulus type	11.547	0.000^∗∗∗^	0.191

*Note.* ^*∗*^ *p* < 0.05, ^*∗∗*^*p* < 0.01, and ^*∗∗∗*^*p* < 0.001.

**Table 4:** Summary of repeated measure ANOVA for anger-neutral pairs—time and stimulus type as within factors on gaze duration to anger-related information.

	*F*	*p*	*η* ^2^
Time	49.90	0.000^∗∗∗^	0.505
Stimulus type	30.19	0.000^∗∗∗^	0.381
Time ∗ stimulus type	5.65	0.000^∗∗∗^	0.103

*Note.* ^*∗*^ *p* < 0.05, ^*∗∗*^*p* < 0.01, and ^*∗∗∗*^*p* < 0.001.

**Table 5:** Means/SD and *t*-test results of the gaze durations for pain- and anger-related stimuli and neutral stimuli on six different time points.

	Stimulus		*t*	*p*
	Pain, M (SD)	Neutral, M (SD)		
Time 1	0.195 (0.039)	0.206 (0.039)	−2.608	0.021
Time 2	0.302 (0.048)	0.229 (0.052)	5.491	0.000^*∗*^
Time 3	0.310 (0.065)	0.260 (0.066)	5.627	0.000^*∗*^
Time 4	0.308 (0.055)	0.273 (0.057)	3.891	0.000^*∗*^
Time 5	0.314 (0.054)	0.291 (0.061)	3.365	0.001^*∗*^
Time 6	0.304 (0.060)	0.279 (0.061)	3.322	0.002^*∗*^
	Anger, M (SD)	Neutral, M (SD)	*t*	*p*
Time 1	0.193 (0.042)	0.202 (0.053)	−1.257	0.215
Time 2	0.296 (0.065)	0.228 (0.076)	5.054	0.000^*∗*^
Time 3	0.302 (0.052)	0.281 (0.063)	2.112	0.040
Time 4	0.309 (0.060)	0.287 (0.063)	1.998	0.051
Time 5	0.313 (0.062)	0.288 (0.064)	2.207	0.032
Time 6	0.306 (0.072)	0.269 (0.074)	3.455	0.001^*∗*^

Bonferroni-corrected *α*' = 0.008.^*∗*^

**Table 6:** Summary of repeated measure ANCOVA—pain bias score-anger bias score pair as a within factor and psychological factors including pain catastrophizing as covariates.

	M (SD)	*F*	*p*	*η* ^2^
Bias score for pain	0.416 (0.346)			
Bias score for anger	0.370 (0.344)			
PA bias score		3.15	0.082	.062
PA bias score ∗ PCS		6.77	0.012^∗^	0.131
PA bias score ∗ STAT_T		0.410	0.525	0.009
PA bias score ∗ STAT_S		0.013	0.910	0.000
PA bias score ∗ CES_D		2.56	0.116	0.054

*Note.* PA bias score = bias score for pain-neutral pairs vs. bias score for anger-neutral pairs, PCS = pain catastrophizing scores, STAT_T = trait anxiety scores, STAT_S = state anxiety scores, CES_D = depression scores; ^*∗*^*p* < 0.05, ^*∗∗*^*p* < 0.01, and ^*∗∗∗*^*p* < 0.001.

## Data Availability

The data used to support the findings of this study are available from the corresponding author upon request.
